# Silencing SCAMP1-TV2 Inhibited the Malignant Biological Behaviors of Breast Cancer Cells by Interaction With PUM2 to Facilitate INSM1 mRNA Degradation

**DOI:** 10.3389/fonc.2020.00613

**Published:** 2020-05-27

**Authors:** Wei Tao, Jun Ma, Jian Zheng, Xiaobai Liu, Yunhui Liu, Xuelei Ruan, Shuyuan Shen, Lianqi Shao, Jiajia Chen, Yixue Xue

**Affiliations:** ^1^Department of Neurobiology, School of Life Sciences, China Medical University, Shenyang, China; ^2^Key Laboratory of Cell Biology, Ministry of Public Health of China, China Medical University, Shenyang, China; ^3^Key Laboratory of Medical Cell Biology, Ministry of Education of China, China Medical University, Shenyang, China; ^4^Department of Neurosurgery, Shengjing Hospital of China Medical University, Shenyang, China; ^5^Liaoning Clinical Medical Research Center in Nervous System Disease, Shenyang, China; ^6^Key Laboratory of Neuro-Oncology in Liaoning Province, Shenyang, China

**Keywords:** RNA binding protein (RBP), SCAMP1-TV2, breast cancer, mRNA degradation, lncRNA, PUM2

## Abstract

**Background:** Molecular-targeted therapy plays an important role in the combined treatment of breast cancer. Long noncoding RNA (LncRNA) plays a significant role in regulating breast cancer progression. The present study is to reveal the potential roles and molecular mechanism that the secretory carrier-associated membrane protein 1-transcript variant 2 (SCAMP1-TV2) has in breast.

**Methods:** Cell Counting Kit-8 (CCK-8), RNA Immunoprecipitation (RIP), and RNA pull-down assays were employed to determine the interactions between SCAMP1-TV2 and Pumilio RNA binding family member 2 (PUM2). The luciferase reporter assays and chromatin immunoprecipitation (ChIP) assays were used to get to know the effect of human insulinoma-associated 1 (INSM1) directly on the SAM and SH3 domain containing 1 (SASH1) promoter.

**Results:** Silenced SCAMP1-TV2 inhibited the proliferation, migration, and invasion of breast cancer cells, and promoted cell apoptosis. Meanwhile, SCAMP1-TV2 downregulation decreased its binding to PUM2 and increased the binding of PUM2 to INSM1 messenger RNA (mRNA), thus promoting the degradation of INSM1 mRNA. Silencing INSM1 decreased its inhibitory effect on SASH1 transcription and inhibited the phosphatidylinositol 3-kinase (PI3K)/protein kinase B (AKT) signaling pathway. The xenograft tumor growth in a nude mice was significantly inhibited by the silencing of SCAMP1-TV2 in combination with the overexpression of PUM2.

**Conclusions:** SCAMP1-TV2/PUM2/INSM1 pathway plays an important role in regulating the biological behavior of breast cancer cells.

## Background

As the second common cancer in the world, breast cancer comprises 25% of all the female cancers. The survival of breast cancer patients has been significantly increased in recent years by improvements in surgical treatment, radiotherapy, chemotherapy, and endocrine therapy. However, the efficacy is not yet ideal for triple negative breast cancer (1). At present, molecule-targeted therapy is a hot topic in the treatment of breast cancer (2).

Long noncoding RNAs (lncRNAs), as RNA transcripts >200 nt without protein coding function, are important in regulating the occurrence and development of various tumors (3). Various lncRNAs have become important biological markers with regard to the diagnosis, treatment efficacy, and prognosis of tumors. LncRNA can regulate tumor occurrence and development at transcriptional, posttranscriptional, and epigenetic levels (4). As a transcript of SCAMP1 gene, lncRNA *Homo sapiens* secretory carrier-associated membrane protein 1, transcript variant 2 (SCAMP1-TV2; GenBank, NR_110885.1) cannot be translated into protein, and the expression and effects of SCAMP1-TV2 in breast cancer have not been reported. Gene expression is able to be regulated at the posttranscriptional level by RNA-binding proteins (RBPs), which should be play an important role in the occurrence and development of tumors. Pumilio RNA binding family member 2 (PUM2) belongs to a PUF family of RBPs. PUM2 can bind with 750 unique messenger RNA (mRNA) targets in humans and plays a critical role in brain development and the maintenance of stem cells (5). Studies completed in recent years have shown that PUM2 has an important regulatory effect on several solid tumors and soft tissue malignant tumors (6, 7). However, the expression and role played by PUM2 in breast cancer have not yet been reported. LncRNAs serve as a “molecular sponge” or “molecular scaffold” for RBP to regulate the expression of downstream genes (8, 9). PTBP3 protein can recruit abundant lnc-nuclear enriched abundant transcript 1 (NEAT1) splicing variants to promote hepatocellular carcinoma (7). Maternally expressed 3 (MEG3) serves as a guide RNA scaffold by recruit polypyrimidine tract binding protein 1 (PTBP1) to destabilize Shp mRNA to cause cholestasis (9). Based on the prediction with the bioinformatics software in this study, there was a binding site between SCAMP1-TV2 and PUM2, which indicates that SCAMP1-TV2 may play its biological role by binding with PUM2. After the prediction using the bioinformatics software, we found out that the insulinoma-associated 1 (INSM1) expression was downregulated more significantly by PUM2 overexpression.

Human INSM1 gene is located at chromosome 20p11.2, and it encodes 510 amino acids (10). INSM1 is mainly expressed in neuroendocrine tissues at some development stages, and especially expressed at a high level in central nervous tissues, pancreatic islets, and neuroendocrine tumors (11). In addition, INSM1 is expressed during the development of endocrine organs, e.g., thyroids, adrenal glands, and thymus glands. INSM1 is highly expressed in medullary thyroid carcinoma, small cell lung cancer, and cervical carcinoma, and can regulate the biological behaviors of tumor cells (12–14). At present, the expression and potential regulatory effects of INSM1 in breast cancer currently remains unclear. In this study, the endogenous expression of SCAMP1-TV2, PUM2, and INSM1 in breast cancer tissues and cells was determined. Then, further investigation was done on the relationship between these molecules and their effects on the biological behaviors of breast cancer cells, so as to reveal the novel mechanism for the morbidity and progress of breast cancer, and offer another therapy for curing the breast cancer.

## Methods

### Human-Tissue Samples

Human breast cancer specimens and their nearby tissues were from such cancer patients who got surgery from 2015 to 2017 at the Breast Surgeons Department, the First Affiliated Hospital, Jinzhou Medical University. By following the WHO classification of tumors in breast cancer (2012, 4th edition), the breast cancer specimens were divided into two categories according to immunohistochemical results: luminal A [R(+)PR(+)Her-2(–)Ki-67 <14%] and triple negative [ER(–)PR(–)Her-2(–)] breast cancer by two competent clinical pathologists. The methods applied in our study were approved by the Institutional Review Board at The First Affiliated Hospital, Jinzhou Medical University. The consent was given by all the related patients, and the study was approved by the Ethics Committee of The First Affiliated Hospital of Jinzhou Medical University.

### Cell Culture

The human MCF-10A, MCF-7, and MDA-MB-231 cell lines were purchased from Chinese Academy of Medical Sciences (Shanghai, People's Republic of China). Human embryonic kidney (HEK) 293T cell lines were purchased from the Shanghai Institutes of Biological Sciences Cell Resource Center. MCF-10A cells were cultured in Dulbecco's modified Eagle's medium (DMEM)/F12 supplemented with 5% horse serum, 20 ng/ml epidermal growth factor (EGF), 0.5 mg/ml hydrocortisone, 100 ng/ml cholera toxin, and 10 μg/ml insulin. MCF-7 and HEK-293T cells were cultured in DMEM/high glucose supplemented with 10% fetal bovine serum (FBS). MDA-MB-231 cells were cultured in L15 medium supplemented with 10% FBS. All cells were maintained in a humidified incubator at 37°C with 5% CO_2_.

### Reverse Transcription and Quantitative Real-Time PCR

Total RNA was extracted from the breast cancer and adjacent tissues, as well as MCF-7 and MDA-MB-231 cells. Using Trizol reagent (Life Technologies Corporation, Carlsbad, CA, USA). The RNA concentration and its quality were detected at the 260/280 nm ratio using a Nanodrop spectrophotometer (ND-100). The complementary DNA (cDNA) was generated using a High Capacity cDNA Reverse Transcription Kit (Applied Biosystems, CA, USA). Quantitative real-time PCR (qRT-PCR) was performed using Two-Step SYBR PrimeScript RT-PCR Kit (Takara Bio, Inc, Japan) for the assays of SCAMP1-TV2, PUM2, INSM1, and glyceraldehyde 3-phosphate dehydrogenase (GAPDH). For the primers used, refer to Supplementary Table 1. All qRT-PCR analyses were done using the 7500 Fast Real-Time PCR System (Applied Biosystems). Expressions were normalized to endogenous controls, and the relative quantification (2^−ΔΔ*Ct*^) method was used for fold change calculation.

### Lentiviral Vector Construction and Infection

Short hairpin RNAs directed against SCAMP1-TV2, INSM1, and SAM and SH3 domain containing 1 (SASH1) were, respectively, ligated into the pLV-u6-gfp-Puro lentiviral vector (Hanbio Biotechnology, Shanghai, China). Short hairpin RNA directed against PUM2 was ligated into the pLV-u6-red-bsd lentiviral vector (Hanbio Biotechnology, Shanghai, China). Target sequences are listed in Supplementary Table 2. The INSM1 and SASH1 coding sequence (CDS) were ligated into the pLV-cmv-red-bsd lentiviral vector (Hanbio Biotechnology, Shanghai, China). The PUM2 coding sequence (CDS) was ligated into the pLV-cmv-gfp-Puro lentiviral vector (Hanbio Biotechnology, Shanghai, China). Lentivirus was harvested 48 h after the lentiviral vectors or the empty lentiviral vectors (negative control, NC) cotransfected with the packaging vectors into human breast cancer cell lines MCF-7 and MDA-MB-231. Cells were then infected with the lentivirus to obtain the sh-SCAMP1-TV2, sh-PUM2, sh-INSM1, and sh-SASH1 cells, or PUM2, INSM1, and SASH1 overexpressing cells. The overexpression and knockdown efficiency are shown in Supplementary Figure 1.

### Cell Proliferation Assay

Cell Counting Kit-8 assays (CCK-8, Dojin, Japan) were performed to determine the proliferation of MCF-7 and MDA-MB-231 breast cancer cells. After the transfection, cells were seeded in 96-well plates in a density of 2,000 cells per well. After 72 h, 10 μl of CCK-8 solution was added into each well and incubated for 2 h at 37°C. The absorbance was measured at 450 nm with the SpectraMax M5 microplate reader.

### Quantization of Apoptosis by Flow Cytometry

Using the Annexin 7AAD/PE staining (Southern Biotech, Birmingham the AL, USA), cell apoptosis was quantified. The cells, after being rinsed for twice with PBS and centrifugalized, were resuspended in Annexin-V-7AAD/PE binding buffer and stained with Annexin 7AAD/PE based on the manufacturer's instructions. The cells were then analyzed by flow cytometry (FACScan, BD Biosciences) to get the apoptotic fractions.

### Cell Migration and Invasion Assay

Chambers (24-well) with 8-μm pore size (Costar, Corning, NY, USA) were accepted to test the migration and invasion of MCF-7 and MDA-MB-231. Such tests were done in a way that the cells were resuspended in 100 μl serum-free medium at a density of 10^5^/ml and seeded in the upper chamber [or chambers were precoated with 500 ng/ml Matrigel solution (BD, Franklin Lakes, NJ)]. Six hundred microliters medium of 10% FBS was placed in the lower chamber for 48 h; the cells on the upper membrane surface were then physically wiped out with a cotton swab. Cells that had migrated or invaded to the lower side of the membrane were fixed with methanol and stained with 10% Giemsa. Then, five random fields were chosen to count the cells under a microscope, and the photographs were taken.

### Reporter Vector Construction and Dual Luciferase Reporter Assays

The INSM1 binding sites in the SASH1 promoter were predicted by the JASPAR software. For the reporter constructs, the SASH1 promoter regions (2,000 to +200 bp) were amplified from human genomic DNA by PCR. Furthermore, putative INSM1 binding sites in the PCR conducts were deleted one after another. The PCR products were subcloned into the pGL3 vector (Promega, Madison, WI, USA) upstream of a luciferase gene. The human full-length INSM1 gene was cloned in pEX3 (pGCMV/MCS/Neo) plasmid vector (GenePharma, Shanghai, China). HEK293T cells were cotransfected with the pGL3 vector with either full-length or deleted promoter regions and pEX3-INSM1 or pEX3 empty vector using Lipofectamine 3000. For the analysis on the luciferase activity, refer to the previous description.

### Western Blot Assay

Using radioimmunoprecipitation assay (RIPA) buffer containing 50 mM HEPES, 1 mM ethylenediaminetetraacetic acid (EDTA) (pH 8.0) on ice, the total protein was extracted from frozen cells. The samples were centrifuged for 40 min at 17,000 rpm and 4°C, and the protein concentration of the supernatant extracts was obtained by bicinchoninic acid (BCA) protein assay kit (Beyotime, Shanghai, China). They were subjected to sodium dodecyl sulfate–polyacrylamide gel electrophoresis (SDS-PAGE) and electrophoretically transferred to polyvinylidene fluoride (PVDF) membranes. Then, such membranes were incubated in Tris-buffered saline containing 5% nonfat milk for 2 h at room temperature and then incubated for 18 h with primary antibodies as follows: PUM2 (1:2,000, Abcam, Cambridge, MA, USA), INSM1 (1:500, Santa Cruz Biotechnology, Santa Cruz, CA, USA), SASH1 (1:1,000, Abcam, Cambridge, MA, USA), and GAPDH (1:1,000, Santa Cruz Biotechnology, Santa Cruz, CA, USA). Horseradish peroxidase (HRP)-linked antimouse inmmunoglobulin G (IgG) and HRP-linked antirabbit IgG antibodies were used as secondary antibodies. The signals were quantified using FluorChem 2.0 software (Alpha Innotech, San Leandro, CA, USA).

### Immunohistochemistry

All paraffin-embedded specimens were sliced into serial 4-μm sections and labeled with primary antibodies anti-PUM2 (1:50, Abcam, Cambridge, MA, USA), anti-INSM1 (1:50, Santa Cruz Biotechnology, Santa Cruz, CA, USA), followed by incubation with the biotinylated secondary antibody included in the immunohistochemical labeling kit (KIT-7780, MaxVision, Fu Zhou, China). Nuclei were counterstained with hematoxylin. To evaluate the expression levels of PUM2 and INSM1, immunostained human breast tissues were judged by two pathologists.

### RNA Immunoprecipitation Assay

RNA immunoprecipitation (RIP) was performed using a Magna RNA-binding protein immunoprecipitation kit (Millipore, Billerica, MA, USA) based on the instructions from the manufacturer. Whole-cell lysate was incubated with RIP buffer containing magnetic beads conjugated with human anti-PUM2 antibody or with negative control normal rabbit IgG. Samples were incubated with Proteinase K, and then, the immunoprecipitated RNA was isolated. The RNA concentration was measured by a spectrophotometer (NanoDrop, Thermo Scientific, Waltham, MA, USA), and the RNA quality was assessed using a bioanalyzer (Agilent, Santa Clara, CA, USA). Furthermore, purified RNAs were extracted and then analyzed by RT-PCR to demonstrate the presence of the binding targets.

### RNA Pull-Down Assay

The interaction between SCAMP1-TV2 and PUM2 (or PUM1) was examined using Pierce Magnetic RNA-Protein Pull-Down Kit (Thermo Fisher) according to the manufacturer's protocols. Biotin-labeled SCAMP1-TV2 or antisense RNA was coincubated with protein extract of MCF-7 or MDA-MB-231 cells and magnetic beads. Low speed centrifugation was used to generate the bead–RNA–protein complex. With the bead compound washing with Handee spin columns, it was boiled in SDS buffer, and the retrieved protein was detected using the GAPDH control.

### Chromatin Immunoprecipitation Assay

Simple chromatin immunoprecipitation (ChIP) Enzymatic Chromatin IP Kit (Cell Signaling Technology, Danvers, MA, USA) was used for ChIP assays according to the manufacturer's protocol. Briefly, cells were crosslinked with 1% formaldehyde and collected in lysis buffer. Chromatin was then digested with micrococcal nuclease. Immunoprecipitation was incubated with 3 μg of anti-INSM1 antibody or normal rabbit IgG followed by immunoprecipitation with protein G agarose beads during an overnight incubation at 4°C with gentle shaking. As an input reference, 2% of the volume was removed before incubation with the antibody and stored at −20°C. The ChIP DNA was reverse crosslinked with 5 mol/L NaCl and Proteinase K and then purified. Immunoprecipitated DNA was amplified by PCR using primers, which are listed in Supplementary Table 3.

### Tumor Xenografts in Nude Mice

The animal experiment was done in strict conformance with the Plan requested to be followed by the Animal Protection Committee of China Medical University. The stable-expression MCF-7 and MDA-MB-231 cells were used for *in vivo* study. Four-week-old BALB/C athymic nude mice were obtained from the National Laboratory Animal Center (Beijing, People's Republic of China). The animals received autoclaved food and water *ad libitum* during the study. The nude mice were divided into five groups: control group (only MCF-7 or MDA-MB-231), sh-SCAMP1-TV2-NC combined with PUM2-NC group (sh-SCAMP1-TV2-NC stable expression MCF-7 or MDA-MB-231 cells infected with PUM2-NC stable-overexpression viruses), sh-SCAMP1-TV2 group (SCAMP1-TV2 knockdown stable-expression MCF-7 or MDA-MB-231 cells), PUM2 group (PUM2 stable-overexpression MCF-7 or MDA-MB-231 cells), sh-SCAMP1-TV2 combined with PUM2 group (sh-SCAMP1-TV2 stable-expression MCF-7 or MDA-MB-231 cells infected with PUM2 stable-overexpression viruses). A total of 3 × 10^5^ cells were injected into the fourth mammary fat pad of the mice. Tumor volume was measured every 5 days, and the volume was calculated by the formula: volume (mm^3^) = length × width^2^/2.

### Statistical Analysis

Data were presented as mean ± SD. All statistical analyses were evaluated by SPSS 18.0 statistical software with the Student's *t*-test or one-way analysis of variance (ANOVA). Differences were considered to be significant when *P* < 0.05.

## Results

### Knockdown of SCAMP1-TV2 Inhibited the Malignant Biological Behaviors of Breast Cancer Cells

**Figure 1:**
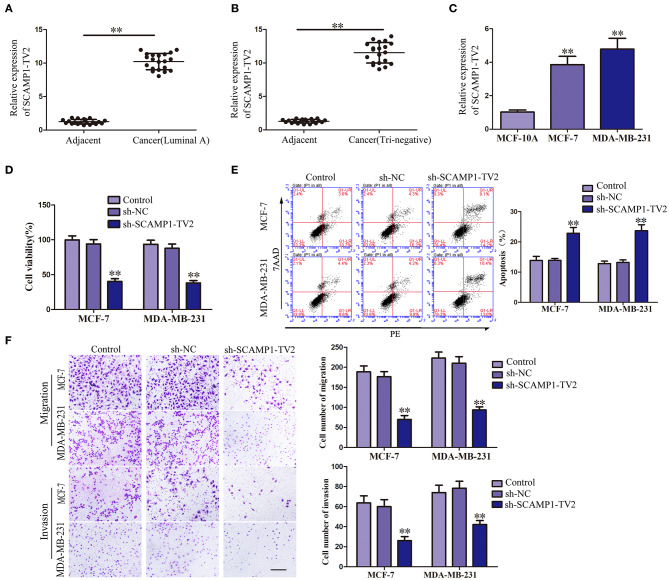
Secretory carrier-associated membrane protein 1-transcript variant 2 (SCAMP1-TV2) knockdown inhibited the malignant behaviors of breast cancer cells. **(A)** Expression levels of SCAMP1-TV2 in breast cancer (luminal A) tissues and adjacent tissues. **(B)** Expression levels of SCAMP1-TV2 in breast cancer (triple negative) tissues and adjacent tissues. Data are presented as the mean ± SD (*n* = 20, each group), **P < 0.01 vs. adjacent tissues. **(C)** Expression levels of SCAMP1-TV2 in MCF-10A, MCF-7, and MDA-MB-231. Data are presented as the mean ± SD (*n* = 3, each group). **P < 0.01 vs. MCF-10A group. **(D)** Cell Counting Kit-8 (CCK-8) assay was applied to evaluate the effect of SCAMP1-TV2 knockdown on the proliferation of breast cancer cells. **(E)** Flow cytometry analysis of breast cancer cells with the SCAMP1-TV2 knockdown. **(F)** Quantification of migration and invasion cells with the SCAMP1-TV2 knockdown. Representative images and accompanying statistical plots were presented (scale bar = 100 μm). Data are presented as the mean ± SD (*n* = 3, each group), **P < 0.01 vs. sh-NC group.

In order to evaluate the effects of SCAMP1-TV2 in breast cancer, the expression level of SCAMP1-TV2 was first assessed in breast cancer tissues and cells. As compared with paracancerous tissues, the expression of SCAMP1-TV2 was significantly increased in luminal A and triple negative breast cancer tissues (Figures 1A,B). Compared with MCF-10A cells, the expression of SCAMP1-TV2 was significantly increased in MCF-7 and MDA-MB-231 cells (Figure 1C and Supplementary Figure 2). To further investigate the function of SCAMP1-TV2, stable-transfected sh-SCAMP1-TV2 cells were established, and we confirmed the knockdown efficiency of SCAMP1-TV2 (Supplementary Figure 3). As compared with sh-NC cells, the viability, migration, and invasion of sh-SCAMP1-TV2 cells were significantly decreased (Figures 1D,F), whereas the apoptosis was markedly increased (Figure 1E).

### Overexpression of PUM2 Inhibited the Malignant Biological Behaviors of Breast Cancer Cells

**Figure 2:**
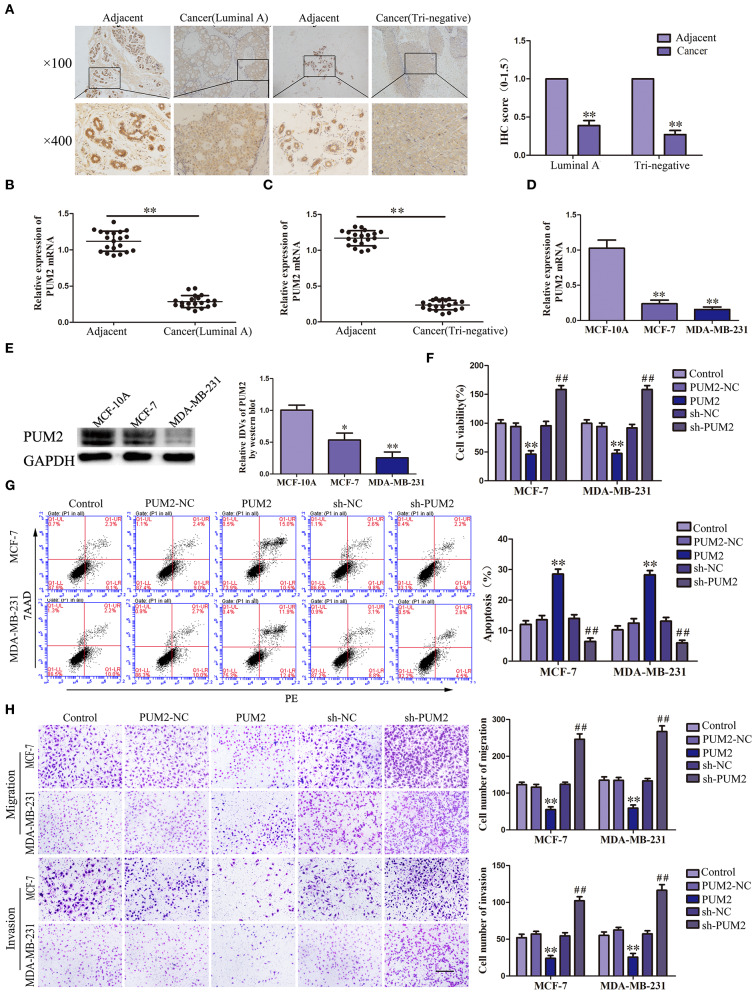
Pumilio RNA binding family member 2 (PUM2) overexpression inhibited the malignant behaviors of breast cancer cells. **(A)** Immunohistochemistry of PUM2 protein in luminal A and triple-negative breast cancer tissues. **(B)** Expression levels of PUM2 messenger RNA (mRNA) in breast cancer (luminal A) tissues and adjacent tissues. **(C)** Expression levels of PUM2 mRNA in breast cancer (triple negative) tissues and adjacent tissues. Data are presented as the mean ± SD (*n* = 20, each group), **P < 0.01 vs. adjacent tissues. **(D)** Expression levels of PUM2 in MCF-7 and MDA-MB-231 breast cancer cells. Data are presented as the mean ± SD (*n* = 3, each group), **P < 0.01 vs. MCF-10A group. **(E)** PUM2 protein expression in MCF-7and MDA-MB-231 breast cancer cells. Data are presented as the mean ± SD (*n* = 3, each group), *P < 0.05 and **P < 0.01 vs. MCF-10A. **(F)** Cell Counting Kit-8 (CCK-8) assay was applied to evaluate the effect of PUM2 on the proliferation of breast cancer cells. **(G)** Flow cytometry analysis of breast cancer cells with PUM2 overexpression and knockdown. **(H)** Quantification of migration and invasion cells with PUM2 overexpression and knockdown. Representative images and accompanying statistical plots were presented (scale bar = 100 μm). Data are presented as the mean ± SD (*n* = 3, each group), **P < 0.01 vs. PUM2-NC group, ^##^*P* < 0.01 vs. sh-NC group.

The bioinformatics software RBPMAP and Starbase 2.0 predicted that PUM2 can bind to SCAMP1-TV2, indicating that SCAMP1-TV2 may require PUM2 for its function. Therefore, we first detected the expression of PUM2 in breast cancer tissues and cells. PUM2 was mainly distributed in the cytoplasm, and luminal A and triple negative breasts cancer tissues had decreased PUM2 expression compared to paracancerous tissues (Figure 2A). As shown in Figures 2B,C, the mRNA expression of PUM2 was also significantly decreased in breast cancer tissues of luminal A type and triple negative type compared to paracancerous tissues. As compared with MCF-10A cells, the mRNA and protein expression levels of PUM2 were markedly lower in MCF-7 cells and MDA-MB-231 cells (Figures 2D,E). Stable-transfected PUM2 and sh-PUM2 cells were established, and then, the changes in the biological behaviors of cells were detected. As compared with PUM2-NC group, the viability, migration, and invasion of MCF-7 and MDA-MB-231 cells in PUM2 groups were significantly decreased, whereas the apoptosis was increased. As compared with sh-NC group, the contrary results were observed in sh-PUM2 groups (Figures 2F–H).

### PUM2 Bound to SCAMP1-TV2 in a Targeted Manner

**Figure 3:**
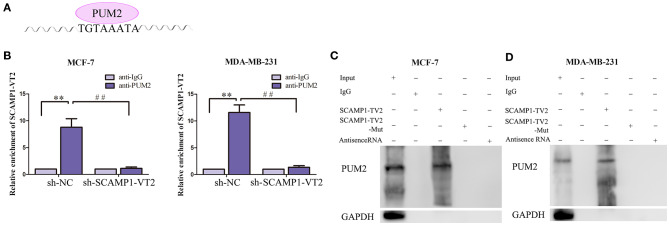
There was targeted binding between secretory carrier-associated membrane protein 1-transcript variant 2 (SCAMP1-TV2) and Pumilio RNA binding family member 2 (PUM2). **(A)** The binding site of PUM2 on SCAMP1-TV2. **(B)** Cellular lysates from MCF-7 and MDA-MB-231 cells were used for RNA immunoprecipitation with antibody against PUM2; SCAMP1-TV2 expression levels were detected using quantitative real-time PCR (qRT-PCR). Data were presented as mean ± SD (*n* = 3, each group), **P < 0.01 vs. sh-NC+anti-IgG group, ^##^*P* < 0.01 vs. sh-NC+anti-PUM2 group. **(C,D)** Detection of PUM2 using Western blot analysis in the sample pulled down by biotinylated SCAMP1-TV2 probe from MCF-7 and MDA-MB-231cells. The mutant-type SCAMP1-TV2 (SCAMP1-TV2-Mut) was three binding site mutant. Data were presented as mean ± SD (*n* = 3, each group).

In order to validate the interaction between SCAMP1-TV2 and PUM2, RIP assay and RNA pull-down assay were performed. As compared with the IgG immunoprecipitate, SCAMP1-TV2 was enriched in the PUM2 immunoprecipitate of MCF-7 and MDA-MB-231 cells. After SCAMP1-TV2 knockdown, such enrichment in PUM2 immunoprecipitate was significantly decreased (Figures 3A,B). The RNA pull-down assay was then conducted to further determine the binding between SCAMP1-TV2 and PUM2. There are three binding sites of PUM2 to SCAMP1-TV2. In MCF-7 and MDA-MB-231 cells, PUM2 was found only in the pull-down samples of sense-strand RNA probe for wild-type SCAMP1-TV2, but not in the antisense-strand RNA probe or mutant-type SCAMP1-TV2 (mutant 3 binding sites) (Figures 3C,D). Additionally, because of PUM1 and PUM2 binding to the similar motif, thus, we also detected whether PUM1 binds to SCAMP1-TV2. We found that there was no binding site between SCAMP1-TV2 and PUM1 (Supplementary Figure 4). These findings suggested that PUM2 bound SCAMP1-TV2 in a targeted way, and this binding was decreased by silencing SCAMP1-TV2.

### Knockdown of INSM1 Inhibited the Malignant Biological Behaviors of Breast Cancer Cells

**Figure 4:**
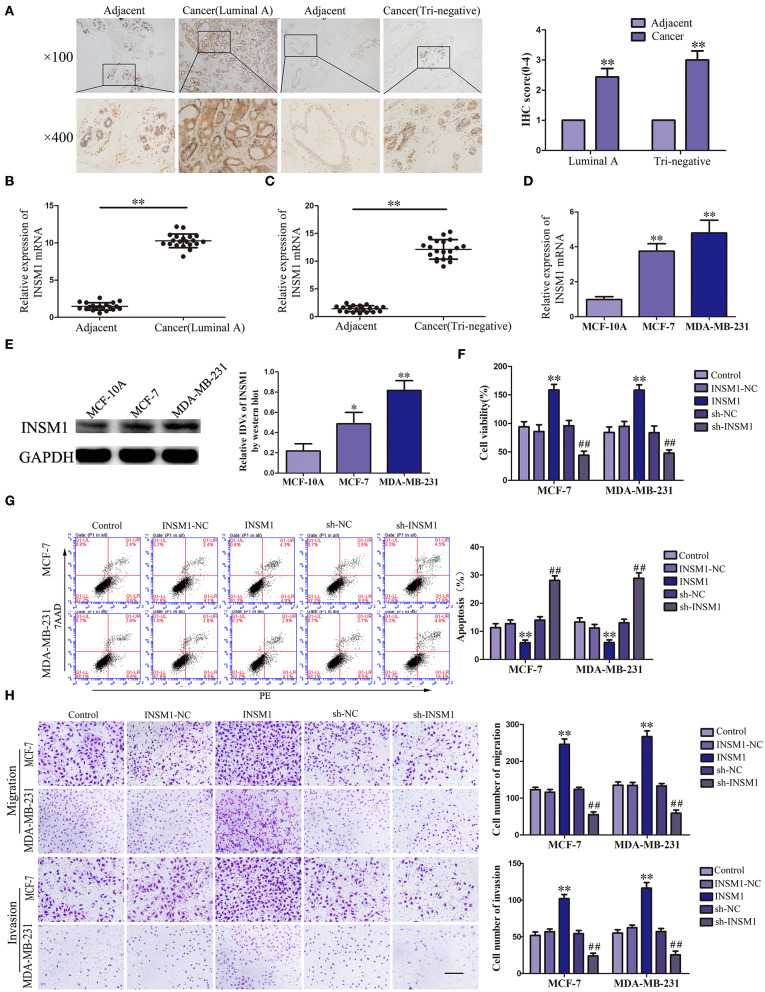
Insulinoma-associated 1 (INSM1) knockdown inhibited the malignant behaviors of breast cancer cells. **(A)** Immunohistochemistry of INSM1 protein in luminal A and triple-negative breast cancer tissues. **(B)** Expression of INSM1 messenger RNA (mRNA) in breast cancer (luminal A) tissues. **(C)** Expression of INSM1 mRNA in breast cancer (triple negative) tissues. Data are presented as the mean ± SD (*n* = 20, each group), **P < 0.01 vs. adjacent tissues. **(D)** Expression of INSM1 mRNA in MCF-7 and MDA-MB-231 breast cancer cells. Data are presented as the mean ± SD (*n* = 3, each group), **P < 0.01 vs. MCF-10A group. **(E)** INSM1 protein expression in MCF-7 and MDA-MB-231 breast cancer cells. Data are presented as the mean ± SD (*n* = 3, each group), *P < 0.05 and **P < 0.01 vs. MCF-10A. **(F)** Cell Counting Kit-8 (CCK-8) assay was applied to evaluate the effect of INSM1 on the proliferation of breast cancer cells. **(G)** Flow cytometry analysis of breast cancer cells with INSM1 overexpression and knockdown (*n* = 3, each group). **(H)** Quantification of migration and invasion cells with INSM1 overexpression and knockdown. Representative images and accompanying statistical plots were presented (scale bar = 100 μm). Data are presented as the mean ± SD (*n* = 3, each group), **P < 0.01 vs. INSM1-NC group, ^##^*P* < 0.01 vs. sh-NC group.

Previous studies have suggested that PUM2 can degrade target mRNA by binding with the 3′-untranslated region (UTR) and thus inhibit the gene expression. In this study, we used the bioinformatics software RBPMAP and Starbase 2.0 to predict potential PUM2 binding sites in 3′-UTR of several mRNAs. Assessment of the mRNA expression in breast cancer cells after PUM2 overexpression showed that the expression of INSM1 was downregulated more significantly by PUM2 overexpression (Supplementary Figure 5). Therefore, we further detected the role of INSM1 in breast cancer. As shown in Figure 4A, the expression of INSM1 was significantly increased in luminal A and triple negative breast cancer tissues compared with paracancerous tissues. The levels of INSM1 mRNA were significantly increased in luminal A and triple negative breast cancer tissues (Figures 4B,C). As compared with MCF-10A cells, the mRNA and protein expression levels of INSM1 were significantly increased in MCF-7 and MDA-MB-231 cells (Figures 4D,E). The stable transfected cells of INSM1 and sh-INSM1 were established, and the changes in the biological behaviors of cells were detected. Compared with INSM1-NC group, the viability, migration, and invasion of MCF-7 and MDA-MB-231 cells in the INSM1 group were significantly increased, whereas the apoptosis rate was significantly decreased. The opposite results were observed in sh-INSM1(–) group compared with the sh-NC group (Figures 4F–H).

### PUM2 Overexpression and SCAMP1-TV2 Knockdown Inhibited the INSM1 Expression

**Figure 5:**
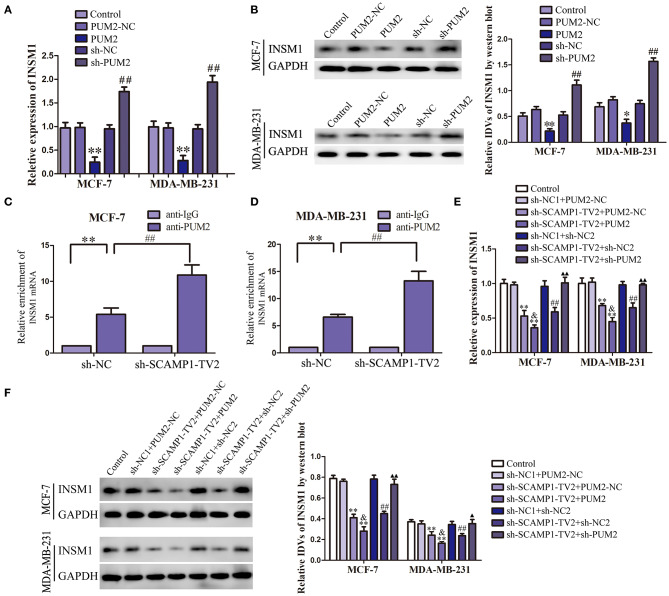
Pumilio RNA binding family member 2 (PUM2) overexpression inhibited insulinoma-associated 1 (INSM1) expression and knockdown of secretory carrier-associated membrane protein 1-transcript variant 2 (SCAMP1-TV2) increased the inhibitory effect of PUM2 on INSM1 expression by binding INSM1 messenger RNA (mRNA). **(A)** The mRNA expression levels of INSM1 regulated by PUM2 overexpression and knockdown. **(B)** The protein expression levels of INSM1 regulated by PUM2 overexpression and knockdown. The integrated density values (IDVs) of INSM1 are shown using glyceraldehyde 3-phosphate dehydrogenase (GAPDH) as an endogenous control. Data are presented as the mean ± SD (*n* = 3, each group), *P < 0.05 and **P < 0.01 vs. PUM2-NC group, ^##^*P* < 0.05 vs. sh-NC group. Cellular lysates from **(C)** MCF-7 and **(D)** MDA-MB-231 cells were used for RNA immunoprecipitation with antibody against PUM2; INSM1 expression levels were detected using quantitative real-time PCR (qRT-PCR). Data were presented as mean ± SD (*n* = 3, each group), **P < 0.01 vs. sh-NC+anti-IgG group, ^##^*P* < 0.01 vs. sh-NC+anti-PUM2 group. The **(E)** mRNA and **(F)** protein expression levels of INSM1 regulated by SCAMP1-TV2 and PUM2. The IDVs of INSM1 are shown using GAPDH as an endogenous control. Data are presented as the mean ± SD (*n* = 3, each group), **P < 0.01 vs. sh-NC1+PUM2-NC group, ^##^*P* < 0.01 vs. sh-NC1+sh-NC2 group, ^&^*P* < 0.05 vs. sh-SCAMP1-TV2+PUM2-NC group, ^▴^*P* < 0.05 and ^▴▴^*P* < 0.01 vs. sh-SCAMP1-TV2+sh-NC2 group.

In order to verify the role of PUM2 in regulating INSM1 expression, the changes in the mRNA and protein expression levels of INSM1 were detected after knockdown or overexpression of PUM2. The mRNA and protein expression of INSM1 in MCF-7 and MDA-MB-231 cells were significantly decreased by the overexpression of PUM2, but markedly increased by the silencing of PUM2 (Figures 5A,B). Subsequently, RIP was performed to assess the binding of NSM1 mRNA to PUM2. As compared with the IgG immunoprecipitate, INSM1 mRNA was enriched in the PUM2 immunoprecipitate of MCF-7 and MDA-MB-231 cells. After the knockdown of SCAMP1-TV2, this enrichment was significantly increased (Figures 5C,D). Furthermore, MCF-7 and MDA-MB-231 cells were double transfected with SCAMP1-TV2 and PUM2 to determine the regulation effects of SCAMP1-TV2 and PUM2 on INSM1 expression, and results showed that the mRNA and protein expression of INSM1 was decreased by SCAMP1-TV2 silencing alone and SCAMP1-TV2 silencing in combination with PUM2 overexpression, and the expression of INSM1 was lower after SCAMP1-TV2 silencing combined with PUM2 overexpression group than in SCAMP1-TV2 silencing alone. In addition, the decreased expression induced by SCAMP1-TV2 silencing was rescued by PUM2 silencing (Figures 5E,F). These results revealed that PUM2 inhibited the expression of INSM1 by binding to its 3′-UTR, and this inhibitory effect was increased by the knockdown of CAMP1-TV2.

### INSM1 Reverses the Effect of PUM2 on the Biological Behavior of Breast Cancer Cells

**Figure 6:**
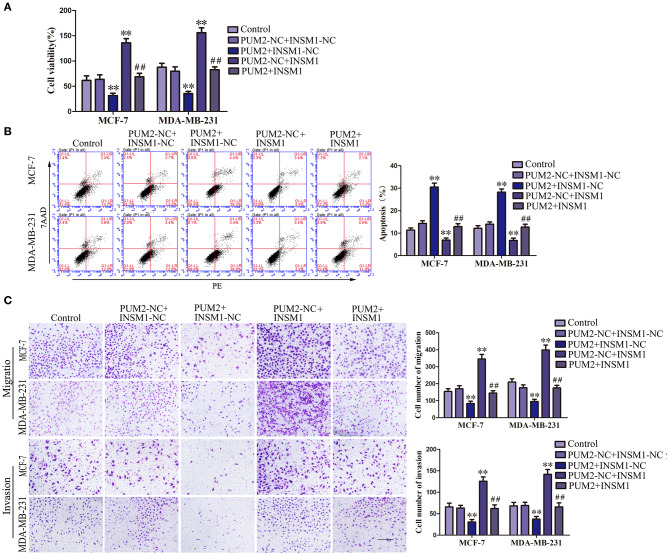
Overexpression of Pumilio RNA binding family member 2 (PUM2) inhibited the malignant behaviors of breast cancer cells by regulating insulinoma-associated 1 (INSM1) expression. **(A)** Cell Counting Kit-8 (CCK-8) assay was applied to evaluate the effect of PUM2 and INSM1 on the proliferation of breast cancer cells. **(B)** Flow cytometry analysis of breast cancer cells with the expression of PUM2 and INSM1 changed. **(C)** Quantification of migration and invasion cells with the expression of PUM2 and INSM1 changed. Representative images and accompanying statistical plots were presented (scale bar = 100 μm). Data are presented as the mean ± SD (*n* = 3, each group), **P < 0.01 vs. PUM2-NC+INSM-NC group, ^##^*P* < 0.01 vs. PUM2+INSM1-NC group.

To confirm whether PUM2 regulates the behavior of breast cancer cells through its regulation of INSM1, MCF-7 and MDA-MB-231 cells were cotransfected with PUM2 and INSM1. As compared to the PUM2-NC+ INSM1-NC group, the viability, migration, and invasion of breast cancer cells were decreased in the PUM2+INSM1-NC group, but the apoptosis rate was increased; the contrary results were found in PUM2–NC+INSM1 group. The inhibitory effects of PUM2 overexpression on the malignant biological behaviors of breast cancer cells was reversed by INSM1 overexpression (Figures 6A–C). These findings demonstrated that INSM1 participated in the regulation of PUM2 on the biological behaviors of breast cancer cells.

### INSM1 Transcriptionally Inhibited SASH1 Expression

**Figure 7:**
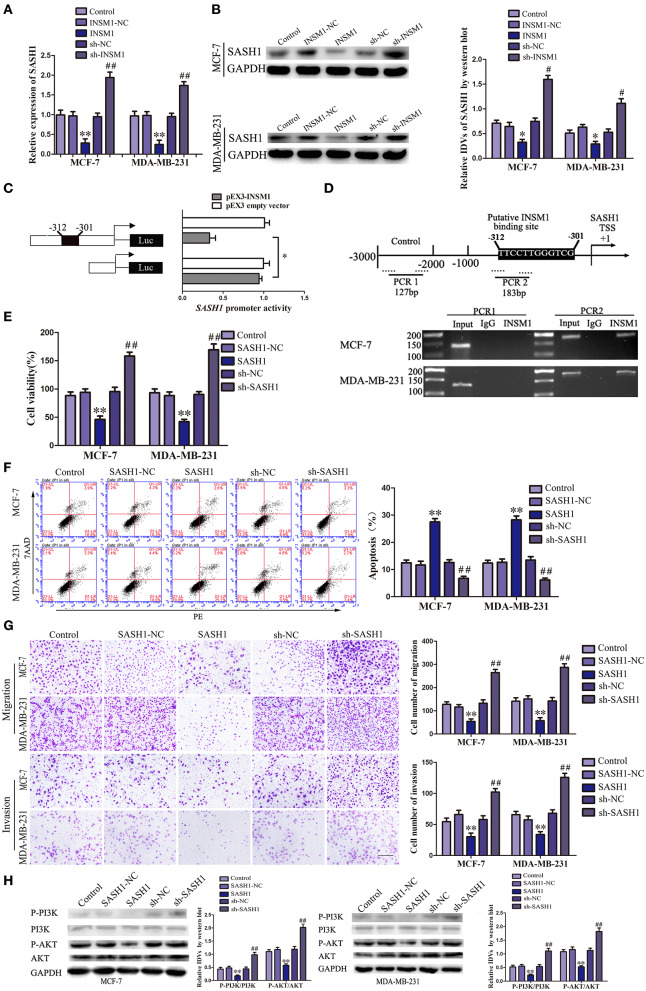
Insulinoma-associated 1 (INSM1) transcriptionally inhibited SAM and SH3 domain containing 1 (SASH1) expression, and SASH1 inhibited the malignant behaviors of breast cancer cells through inhibiting phosphatidylinositol 3-kinase (PI3K)/protein kinase B (AKT) pathway. **(A)** The messenger RNA (mRNA) expression of SASH1 regulated by INSM1. **(B)** Effects of overexpression or knockdown of INSM1 on SASH1 expression. The integrated density values (IDVs) of SASH1 are shown using glyceraldehyde 3-phosphate dehydrogenase (GAPDH) as an endogenous control. Data are presented as the mean ± SD (*n* = 3, each group), *P < 0.05 and **P < 0.01 vs. INSM1-NC group, ^#^*P* < 0.05 and ^##^*P* < 0.05 vs. sh-NC group. **(C)** INSM1 on the promoter activity of SASH1 in HEK 293T cells. The deletion construct on the promoter of INSM1 is shown in the Y-bar. The X-bar shows the promoter activity that has been normalized with the reference vector (pRL-TK) and relative to the activity of pEX3 empty vector. Data are presented as the mean ± SD (*n* = 3, each group). **(D)** INSM1 bound to the promoter of SASH1 in MCF-7 and MA-MB-231 breast cancer cells. Transcription start site (TSS) was designated as +1. Putative INSM1 binding sites are indicated. Immunoprecipitated DNA was amplified by PCR. Normal rat immunoglobulin G (IgG) was used as a negative control. **(E)** Cell Counting Kit-8 (CCK-8) assay was applied to evaluate the effect of SASH1 on the proliferation of breast cancer cells. **(F)** Flow cytometry analysis of breast cancer cells with the expression of SASH1 changed. **(G)** Quantification of migration and invasion cells with the expression of SASH1 changed. Representative images and accompanying statistical plots were presented (scale bar = 100μm). Data are presented as the mean ± SD (*n* = 3, each group), **P < 0.01 vs. SASH1-NC group, ^##^*P* < 0.01 vs. sh-NC group. **(H)** Effects of overexpression or knockdown of SASH1 on the activity of PI3K/AKT pathway. The IDVs of PI3K, P-PI3K, AKT, P-AKT are shown using GAPDH as an endogenous control. Data are presented as the mean ± SD (*n* = 3, each group), **P < 0.01 vs. SASH1-NC group, ^##^*P* < 0.01 vs. sh-NC group.

We detected the gene expression after INSM1 overexpression using gene array (data not shown) and found that SASH1 expression is significantly decreased after INSM1 overexpression. Additionally, a potential INSM1 binding site was found in the promoter region of SASH1 using the bioinformatics software JASPAR, which indicated that INSM1 might regulate the expression level of SASH1 by binding its promoter region. In order to validate the regulation of SASH1 by INSM1, changes in the mRNA and protein levels of SASH1 were detected after INSM1 overexpression or knockdown. The mRNA and protein expressions of SASH1 were significantly inhibited by the overexpression of INSM1 but promoted by the silencing of INSM1 (Figures 7A,B). To verify this hypothesis, dual-luciferase and CHIP assays were performed. As compared with the pEX2 empty vector group, the luciferase activity was significantly decreased in the group transfected with luciferase carrier (with a potential binding site) in the promoter regions of SASH1 and pEX2-INSM1, but restored to the level in the group after the cotransfection with luciferase vector (without a potential binding site) in the promoter regions of SASH1 and pEX2-INSM1 (Figure 7C). In order to further validate the direct binding site between INSM1 and the SASH1 promoter, ChIP assay was conducted. The PCR primers were designed in the upstream and downstream of the predicted binding site, and a negative control primer was designed 1,000 bp upstream of the predicted INSM1 binding site. The results showed that INSM1 bound to the predicted binding site in the SASH1 promoter, but did not bind with the control region (Figure 7D). These findings suggested that SASH1 expression was inhibited by INSM1 at the transcriptional level.

### SASH1 Overexpression Inhibited the Malignant Biological Behaviors of Breast Cancer Cells by Inhibiting the Activity of PI3K/AKT Signaling Pathway

We created stable-transfected cell lines of SASH1 overexpression and knockdown cells, and the changes in the biological behaviors of MCF-7 and MDA-MB-231 cells were evaluated. As compared with SASH1-NC group, the viability, migration, and invasion of cells in SASH1 group were significantly decreased, but the apoptosis rate was markedly increased. The contrary results were found in sh-SASH1 group (Figures 7E–G). Previous studies have identified a role of SASH1 in inhibiting phosphatidylinositol 3-kinase (PI3K)/protein kinase B (AKT) signaling in thyroid and liver cancer (15, 16). To assess whether this regulatory mechanism was also present in the breast cancer cells, the effects of SASH1 overexpression and knockdown on PI3K/AKT signaling were assessed. As compared with the SASH1-NC group, the levels of p-PI3K/PI3K and p-AKT/AKT were both significantly decreased in the SASH1 group and increased in the sh-SASH1 group (Figure 7H). In addition, the levels of p-PI3K/PI3K and p-AKT/AKT were both significantly decreased in sh-SCAPM1-TV2 group of MCF-7 and MDA-MB-231 cells (Supplementary Figure 6). Based on these results, the malignant biological behaviors of breast cancer cells might be inhibited by SASH1 overexpression through the inhibition of PI3K/AKT signaling pathway.

### *In vivo* Tumor Growth Was Inhibited by the Silencing of SCAMP1-TV2 in Combination With the Overexpression of PUM2

**Figure 8:**
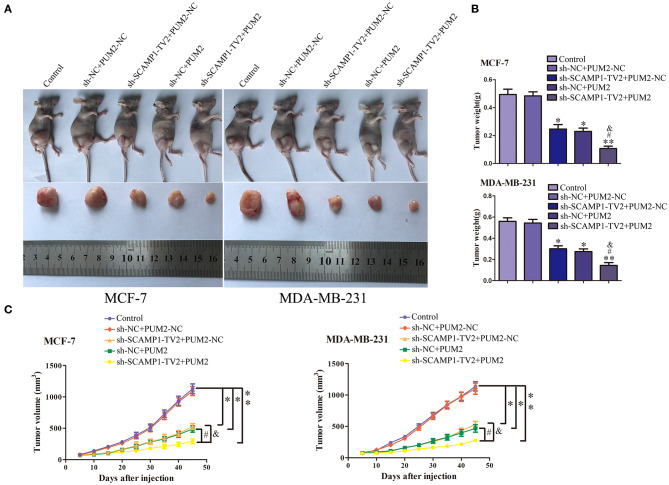
*In vivo* tumor xenografts study. The stable expressing cells were used for the *in vivo* study. **(A)** The nude mice carrying tumors from respective groups were shown. The sample tumor from respective group was shown. **(B)** The tumor was excised and weighed after 40 days. **(C)** Tumor volume was calculated every 5 days after injection, *P < 0.05 or **P < 0.01 vs. sh-NC+PUM2-NC group, ^#^*P* < 0.05 vs. sh-SCAMP1-TV2+PUM2-NC group, ^&^*P* < 0.05 vs. sh-NC+PUM2 group.

In order to further determine the role of SCAMP1-TV2 and PUM2 in breast cancer *in vivo*, the xenograft tumor experiment was performed on the nude mice. The weight and size of xenograft tumors, compared with sh-NC+PUM2-NC group, were both significantly reduced in the sh-SCAMP1-TV2+PUM2-NC, sh-NC+PUM2, and sh-SCAMP1-TV2+PUM2 groups, and the sh-SCAMP1-TV2+PUM2 group had the smallest tumors (Figures 8A,B). In addition, the survival was prolonged in sh-SCAMP1-TV2+PUM2-NC, sh-NC+PUM2, and sh-SCAMP1-TV2+PUM2 groups, and the survival of the sh-SCAMP1-TV2+PUM2 group was the most prolonged (Figure 8C). Therefore, the growth of xenograft tumors in nude mice was most significantly inhibited, and the survival was most prolonged by the silencing of SCAMP1-TV2 in combination with the overexpression of PUM2.

## Discussion

In this study, SCAMP1-TV2 was highly expressed in the samples and breast cancer cells; and its silencing inhibited the viability, migration, and invasion of breast cancer cells, as well as promote the apoptosis. SCAMP1-TV2 bound with PUM2 in a targeted manner. After silencing SCAMP1-TV2, the binding between SCAMP1-TV2 and PUM2 was decreased, the binding between PUM2 and INSM1 mRNA was increased, and INSM1 mRNA was degraded. Thus, the expression level of INSM1 declined. Knockdown of INSM1 weakened the inhibition of INSM1 on SASH1 transcription, and thus, the expression of SASH1 was increased. Meanwhile, the activity of PI3K/AKT signaling pathway was inhibited, and the malignant biological behaviors of breast cancer cells were suppressed. Our study results offer a new experimental basis to investigating the effects of SCAMP1-TV2 in breast cancer.

In recent years, there has been increased attention to the abnormal expression and important regulatory effects of lncRNAs in breast cancer. In the Li et al. study (17), five lncRNAs (e.g., HOTAIR) were used as markers for predicting the recurrence of breast cancer. Peng et al. (18) found that H19 was highly expressed in breast cancer stem cells, and it regulated the stem cell features by the targeted control of let-7. Moreover, various lncRNAs can regulate DNA injury repair and epithelial–mesenchymal transition (EMT) in the mammary cells and thus controlled the progression of breast cancer (19, 20). The lncRNA SCAMP1-TV2 is a transcript variant of SCAMP1 gene, which was named as SCAMP1-TV2 in this study. The expression of SCAMP1-TV2 was significantly upregulated in breast cancer tissues and cells, and silencing SCAMP1-TV2 inhibited the proliferation, migration, and invasion of breast cancer, whereas it promoted the apoptosis. These suggested that SCAMP1-TV2 might act as a carcinogenic factor in luminal A and triple negative breast cancer. So far, there are no reports about the effects of SCAMP1-TV2 in other tumors.

LncRNAs mainly regulate gene expression by acting as a “molecular sponge” of microRNAs (miRNAs) and epigenetic regulators and enhancers. The interaction between lncRNAs and RBPs is an important factor in the occurrence as well as the development of malignant tumors. RBPs can bind lncRNAs and regulate their stability and thus control the biological behaviors of tumor cells. LncRNA NORAD can upregulate the transcripts of Pumilio proteins and plays a role in genomic stability (21). Some studies showed that in a mouse breast cancer model, transforming growth factor beta (TGF-β)-induced EMT was promoted by U2AF65 (an RBP) through Spry1 splicing (22). In hepatocellular carcinoma, the stability of the lncRNA highly upregulated in liver cancer (HULC) was decreased by RBPs through the recruitment of CCR4-NOT complex (23). In addition, lncRNAs can act on RBPs to regulate the downstream garget gene expression. Zhang et al. (9) observed that Shp mRNA was recruited by the lncRNA MEG3, which acts as the molecular scaffold of PTBP1 to promote its degradation. In our study, SCAMP1-TV2 bound to PUM2 in a targeted manner, and silencing SCAMP1-TV2 decreased its binding to PUM2. Similarly, Kim et al. found that lncRNA OIP5-AS1 could competitively bind with HuR to weaken its effects on target mRNA and thus participate in the regulation of proliferating phenotypes (8). As reported by Liu et al., the lncRNA gadd7 bound with TDP-43 to decrease the binding of TDP-43 to the mRNA 3′-UTR of target genes and attenuate the inhibition on the translation process of these genes (24). In this study, PUM2 expression was significantly decreased in breast cancer tissues and expressed at a markedly low level in the MCF-7 and MDA-MB-231 breast cancer cells. PUM2 overexpression inhibited the proliferation, migration, and invasion of breast cancer cells, as well as promoted the apoptosis. This showed that PUM2 serves as a tumor suppresser in breast cancer. In the central nervous system, PUM2 can regulate the expression of voltage-gated sodium ion channel (Nav) and thus control the excitability of neurons (15). In the mammalian neurons, PUM2 can maintain the morphology and function of synapses (16, 25).

In addition, PUM2 can interact with Aurora-A to improve its stability and participate in the regulation of cell mitosis (26). According to some studies, the function of PUM2 is dependent upon tumor type. PUM2 is highly expressed in myeloid leukemia cells and associated with regulating the growth of hematopoietic stem cells and leukemia cells (6). PUM2 also plays a significant role in maintaining the identity of stem cells and tumor progression by negatively regulating mitogen-activated protein kinase (MAPK) (27). Furthermore, PUM2 can bind with mRNA 3′-UTR of downstream molecules in a targeted way to promote their degradation and thus inhibit the gene expression (28–30). This study demonstrated that PUM2 could bind with the 3′-UTR of INSM1 mRNA in a targeted manner and inhibit INSM1 expression. Similarly to our results, PUM2 can bind with SCCRO3 mRNA, and PUM2 overexpression reduced the mRNA expression of SCCRO3, thus influencing the tumor-suppression effects of SCCRO3 (31). In our study, the inhibition on the mRNA and protein expression of INSM1 was increased by the silencing of SCAMP1-TV2 in combination with the overexpression of PUM2. This indicates that the inhibition of PUM2 on INSM1 mRNA is increased by the silencing of SCAMP1-TV2.

INSM1 is a zinc finger transcription factor originally isolated from human pancreatic islets (32). INSM1 is particularly expressed in neuroendocrine tumors, including pituitary tumors, neuroblastoma, and retinoblastoma, and INSM1 is considered as a neuroendocrine marker of high specificity (10, 29, 30). INSM1 is highly expressed in the extraskeletal myxoid chondrosarcoma and is a potential molecular marker for its diagnosis (33). In our study, INSM1 was highly expressed in the luminal A and triple negative breast cancer tissues and cells. Silencing INSM1 significantly inhibited the proliferation, migration, and invasion of the breast cancer cells while promoting the apoptosis, which indicates that INSM1 acts as a carcinogenic factor in breast cancer. Similarly, INSM1 is highly expressed in small cell lung cancer tissues and cells, and INSM1 knockdown inhibits the malignant biological behaviors of small cell lung cancer cells (13). In our study, the INSM1 bound the TTCCTTGGGTCG sequence at −321~−301 in the promoter region of SASH1 and decreased its transcription activity of SASH1, thus inhibiting SASH1 expression.

SASH1 has been regarded as a tumor suppresser in various type of tumors (34–41). This study further demonstrated that SASH1 had low expression in MCF-7 and MDA-MB-231 cells, and SASH1 overexpression inhibited the proliferation, migration, and invasion of these cells while promoting apoptosis. In the study by Zeller et al., SASH1 expression was decreased in 74% of mammary tumors in comparison with normal mammary epithelial tissues (34, 40), which is similar to our study results. Burgess et al. reported that the apoptosis rate was increased for the seven types of breast cancer cells among the eight breast cancer cells with overexpressed SASH1 (42).

**Figure 9:**
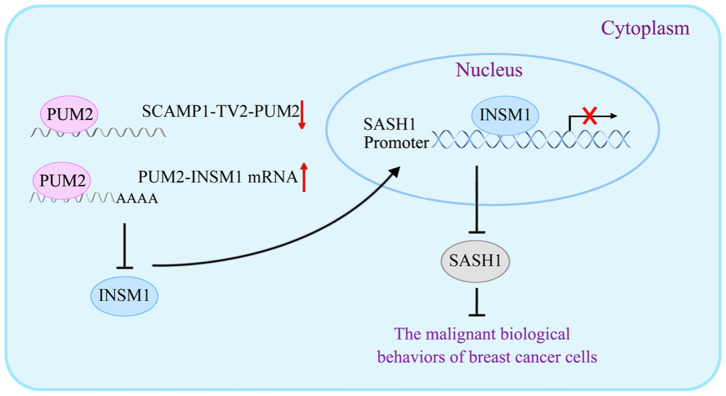
The schematic cartoon of the mechanism underlying the biological behaviors of breast cancer cells by secretory carrier-associated membrane protein 1-transcript variant 2 (SCAMP1-TV2).

In our study, the levels of p-PI3K/PI3K and p-AKT/AKT were significantly decreased by SASH1 overexpression, suggesting that SASH1 blocks the malignant biological behaviors of breast cancer cells through the inhibition of PI3K/AKT signaling pathway. Similarly, SASH1 overexpression inhibits the proliferation and invasion of thyroid cancer cells by suppressing the phosphorylation of PI3K and AKT (43), inhibits the invasion and metastasis of hepatocellular carcinoma cells by suppressing PI3K/AKT signaling pathway (44), and suppresses the TGF-a1-induced EMT of stomach cancer cells by suppressing the phosphorylation of PI3K and AKT (45). For the mechanism underlying the biological behaviors of breast cancer cells by SCAMP1-TV2, refer to Figure 9.

In conclusion, this study first proves that SCAMP1-TV2 is highly expressed in the tissues and cells of breast cancer. Silencing SCAMP1-TV2 inhibits the malignant biological behaviors of breast cancer cells by reducing its binding to PUM2 and further increasing the binding of PUM2 to INSM1 mRNA. This decreased the expression of INSM1 mRNA, which downregulates the inhibitory activity of INSM1 on SASH1 transcription, and inhibited the activity of the PI3K/AKT signaling pathway. Therefore, our results not only validate the important role and mechanism of SCAMP1-TV2 in managing the biological behaviors of breast cancer cells but also provide a new target and a novel mechanism for the molecular targeted treatment of breast cancer.

## Data Availability Statement

All datasets generated for this study are included in the article/Supplementary Material.

## Ethics Statement

Human breast cancer specimens and adjacent tissues were obtained from patients diagnosed with breast cancer who received surgery at the Department of breast surgery of The First Affiliated Hospital, Jinzhou Medical University, from January 2015 to January 2017. The research methods in our study were approved by the Institutional Review Board at The First Affiliated Hospital of Jinzhou Medical University. Informed consents were obtained from all patients, and the study was approved by the Ethics Committee of The First Affiliated Hospital of Jinzhou Medical University.

## Author Contributions

YX: study design, data analysis, writing of the article. WT: performed the experiments, data collection, and data analysis. JM: study design, data collection, and data analysis. JZ: study design and writing of the article. XL: writing of the article. YL: data collection. XR, SS, LS, and JC: data collection. All authors read and approved the final manuscript.

## Conflict of Interest

The authors declare that the research was conducted in the absence of any commercial or financial relationships that could be construed as a potential conflict of interest.

## References

[1] Kandil S Prencipe F Jones S Hiscox S Westwell AD. The discovery of new and more potent chloropyramine (C4) analogues for the potential treatment of invasive breast cancer. Chem Biol Drug Des. (2018) 91:314–21. 10.1111/cbdd.13083 28816016

[2] Tungsukruthai S Petpiroon N Chanvorachote P. Molecular mechanisms of breast cancer metastasis and potential anti-metastatic compounds. Anticancer Res. (2018) 38:2607–18. 10.21873/anticanres.12502 29715080

[3] Wilusz JE Sunwoo H Spector DL. Long noncoding RNAs: functional surprises from the RNA world. Genes Dev. (2009) 23:1494–504. 10.1101/gad.1800909 19571179 PMC3152381

[4] Malek E Jagannathan S Driscoll JJ. Correlation of long non-coding RNA expression with metastasis, drug resistance and clinical outcome in cancer. Oncotarget. (2014) 5:8027–38. 10.18632/oncotarget.2469 25275300 PMC4226665

[5] Zamore PD Williamson JR Lehmann R. The Pumilio protein binds RNA through a conserved domain that defines a new class of RNA-binding proteins. RNA. (1997) 3:1421–33. 9404893 PMC1369583

[6] Naudin C Hattabi A Michelet F Miri-Nezhad A Benyoucef A Pflumio F. PUMILIO/FOXP1 signaling drives expansion of hematopoietic stem/progenitor and leukemia cells. Blood. (2017) 129:2493–506. 10.1182/blood-2016-10-747436 28232582 PMC5429137

[7] Yang X Qu S Wang L Zhang H Yang Z Wang J. PTBP3 splicing factor promotes hepatocellular carcinoma by destroying the splicing balance of NEAT1 and pre-miR-612. Oncogene. (2018) 37:6399–413. 10.1038/s41388-018-0416-8 30068940

[8] Kim J Abdelmohsen K Yang X De S Grammatikakis I Noh JH. LncRNA OIP5-AS1/cyrano sponges RNA-binding protein HuR. Nucleic Acids Res. (2016) 44:2378–92. 10.1093/nar/gkw017 26819413 PMC4797289

[9] Zhang L Yang Z Trottier J Barbier O Wang L. Long noncoding RNA MEG3 induces cholestatic liver injury by interaction with PTBP1 to facilitate shp mRNA decay. Hepatology. (2017) 65:604–15. 10.1002/hep.28882 27770549 PMC5258819

[10] Goto Y De Silva MG Toscani A Prabhakar BS Notkins AL Lan MS. A novel human insulinoma-associated cDNA, IA-1, encodes a protein with “zinc-finger” DNA-binding motifs. J Biol Chem. (1992) 267:15252–7. 1634555

[11] Rosenbaum JN Guo Z Baus RM Werner H Rehrauer WM Lloyd RV. INSM1: a novel immunohistochemical and molecular marker for neuroendocrine and neuroepithelial neoplasms. Am J Clin Pathol. (2015) 144:579–91. 10.1309/AJCPGZWXXBSNL4VD 26386079

[12] De Smaele E Fragomeli C Ferretti E Pelloni M Po A Canettieri G. An integrated approach identifies Nhlh1 and Insm1 as Sonic Hedgehog-regulated genes in developing cerebellum and medulloblastoma. Neoplasia. (2008) 10:89–98. 10.1593/neo.07891 18231642 PMC2213903

[13] Fujino K Motooka Y Hassan WA Ali Abdalla MO Sato Y Kudoh S. Insulinoma-associated protein 1 is a crucial regulator of neuroendocrine differentiation in lung cancer. Am J Pathol. (2015) 185:3164–77. 10.1016/j.ajpath.2015.08.018 26482608

[14] Kuji S Watanabe R Sato Y Iwata T Hirashima Y Takekuma M. A new marker, insulinoma-associated protein 1 (INSM1), for high-grade neuroendocrine carcinoma of the uterine cervix: Analysis of 37 cases. Gynecol Oncol. (2017) 144:384–90. 10.1016/j.ygyno.2016.11.020 27908529

[15] Mee CJ Pym EC Moffat KG Baines RA. Regulation of neuronal excitability through pumilio-dependent control of a sodium channel gene. J Neurosci. (2004) 24:8695–703. 10.1523/JNEUROSCI.2282-04.2004 15470135 PMC6729971

[16] Vessey JP Vaccani A Xie Y Dahm R Karra D Kiebler MA. Dendritic localization of the translational repressor Pumilio 2 and its contribution to dendritic stress granules. J Neurosci. (2006) 26:6496–508. 10.1523/JNEUROSCI.0649-06.2006 16775137 PMC6674044

[17] Li J Wang W Xia P Wan L Zhang L Yu L. Identification of a five-lncRNA signature for predicting the risk of tumor recurrence in patients with breast cancer. Int J Cancer. (2018) 143:2150–60. 10.1002/ijc.31573 29707762 PMC6519083

[18] Peng F Li TT Wang KL Xiao GQ Wang JH Zhao HD. H19/let-7/LIN28 reciprocal negative regulatory circuit promotes breast cancer stem cell maintenance. Cell Death Dis. (2017) 8:e2569. 10.1038/cddis.2016.438 28102845 PMC5386357

[19] Zhang Y He Q Hu Z Feng Y Fan L Tang Z. Long noncoding RNA LINP1 regulates repair of DNA double-strand breaks in triple-negative breast cancer. Nat Struct Mol Biol. (2016) 23:522–30. 10.1038/nsmb.3211 27111890 PMC4927085

[20] Wu W Chen F Cui X Yang L Chen J Zhao J. LncRNA NKILA suppresses TGF-beta-induced epithelial-mesenchymal transition by blocking NF-kappaB signaling in breast cancer. Int J Cancer. (2018) 143:2213–24. 10.1002/ijc.31605 29761481

[21] Lee S Kopp F Chang TC Sataluri A Chen B Sivakumar S. Noncoding RNA NORAD regulates genomic stability by sequestering PUMILIO proteins. Cell. (2016) 164:69–80. 10.1016/j.cell.2015.12.017 26724866 PMC4715682

[22] Rodriguez-Mateo C Torres B Gutierrez G Pintor-Toro JA. Downregulation of Lnc-Spry1 mediates TGF-beta-induced epithelial-mesenchymal transition by transcriptional and posttranscriptional regulatory mechanisms. Cell Death Differ. (2017) 24:785–97. 10.1038/cdd.2017.9 28186499 PMC5423121

[23] Hammerle M Gutschner T Uckelmann H Ozgur S Fiskin E Gross M. Posttranscriptional destabilization of the liver-specific long noncoding RNA HULC by the IGF2 mRNA-binding protein 1 (IGF2BP1). Hepatology. (2013) 58:1703–12. 10.1002/hep.26537 23728852

[24] Liu X Li D Zhang W Guo M Zhan Q. Long non-coding RNA gadd7 interacts with TDP-43 and regulates Cdk6 mRNA decay. EMBO J. (2012) 31:4415–27. 10.1038/emboj.2012.292 23103768 PMC3512391

[25] Zhong J Zhang T Bloch LM. Dendritic mRNAs encode diversified functionalities in hippocampal pyramidal neurons. BMC Neurosci. (2006) 7:17. 10.1186/1471-2202-7-17 16503994 PMC1386695

[26] Dubnau J Chiang AS Grady L Barditch J Gossweiler S McNeil J. The staufen/pumilio pathway is involved in Drosophila long-term memory. Curr Biol. (2003) 13:286–96. 10.1016/S0960-9822(03)00064-2 12593794

[27] Lee MH Hook B Pan G Kershner AM Merritt C Seydoux G. Conserved regulation of MAP kinase expression by PUF RNA-binding proteins. PLoS Genet. (2007) 3:e233. 10.1371/journal.pgen.0030233 18166083 PMC2323325

[28] Spassov DS Jurecic R. Cloning and comparative sequence analysis of PUM1 and PUM2 genes, human members of the Pumilio family of RNA-binding proteins. Gene. (2002) 299:195–204. 10.1016/S0378-1119(02)01060-0 12459267

[29] Breslin MB Zhu M Lan MS. NeuroD1/E47 regulates the E-box element of a novel zinc finger transcription factor, IA-1, in developing nervous system. J Biol Chem. (2003) 278:38991–7. 10.1074/jbc.M306795200 12890672 PMC1236987

[30] Lan MS Breslin MB. Structure, expression, and biological function of INSM1 transcription factor in neuroendocrine differentiation. FASEB J. (2009) 23:2024–33. 10.1096/fj.08-125971 19246490 PMC2704596

[31] Huang G Stock C Bommelje CC Weeda VB Shah K Bains S. SCCRO3 (DCUN1D3) antagonizes the neddylation and oncogenic activity of SCCRO (DCUN1D1). J Biol Chem. (2014) 289:34728–42. 10.1074/jbc.M114.585505 25349211 PMC4263876

[32] Gierl MS Karoulias N Wende H Strehle M Birchmeier C. The zinc-finger factor Insm1 (IA-1) is essential for the development of pancreatic beta cells and intestinal endocrine cells. Genes Dev. (2006) 20:2465–78. 10.1101/gad.381806 16951258 PMC1560419

[33] Yoshida A Makise N Wakai S Kawai A Hiraoka N. INSM1 expression and its diagnostic significance in extraskeletal myxoid chondrosarcoma. Mod Pathol. (2018) 31:744–52. 10.1038/modpathol.2017.189 29327709

[34] Zeller C Hinzmann B Seitz S Prokoph H Burkhard-Goettges E Fischer J. SASH1: a candidate tumor suppressor gene on chromosome 6q24.3 is downregulated in breast cancer. Oncogene. (2003) 22:2972–83. 10.1038/sj.onc.1206474 12771949

[35] Rimkus C Martini M Friederichs J Rosenberg R Doll D Siewert JR. Prognostic significance of downregulated expression of the candidate tumour suppressor gene SASH1 in colon cancer. Br J Cancer. (2006) 95:1419–23. 10.1038/sj.bjc.6603452 17088907 PMC2360597

[36] Chen EG Chen Y Dong LL Zhang JS. Effects of SASH1 on lung cancer cell proliferation, apoptosis, and invasion *in vitro*. Tumour Biol. (2012) 33:1393–401. 10.1007/s13277-012-0387-2 22488244

[37] Lin S Zhang J Xu J Wang H Sang Q Xing Q. Effects of SASH1 on melanoma cell proliferation and apoptosis *in vitro*. Mol Med Rep. (2012) 6:1243–8. 10.3892/mmr.2012.1099 23023727

[38] Nitsche U Rosenberg R Balmert A Schuster T Slotta-Huspenina J Herrmann P. Integrative marker analysis allows risk assessment for metastasis in stage II colon cancer. Ann Surg. (2012) 256:763–71; discussion 771. 10.1097/SLA.0b013e318272de87 23095620

[39] Yang L Liu M Gu Z Chen J Yan Y Li J. Overexpression of SASH1 related to the decreased invasion ability of human glioma U251 cells. Tumour Biol. (2012) 33:2255–63. 10.1007/s13277-012-0487-z 22915266

[40] Sheyu L Hui L Junyu Z Jiawei X Honglian W Qing S. Promoter methylation assay of SASH1 gene in breast cancer. J BUON. (2013) 18:891–8. 24344014

[41] Peng L Wei H Liren L. Promoter methylation assay of SASH1 gene in hepatocellular carcinoma. J BUON. (2014) 19:1041–7. 25536614

[42] Burgess JT Bolderson E Saunus JM Zhang SD Reid LE McNicol AM. SASH1 mediates sensitivity of breast cancer cells to chloropyramine and is associated with prognosis in breast cancer. Oncotarget. (2016) 7:72807–18. 10.18632/oncotarget.12020 27637080 PMC5341945

[43] Sun D Zhou R Liu H Sun W Dong A Zhang H. SASH1 inhibits proliferation and invasion of thyroid cancer cells through PI3K/Akt signaling pathway. Int J Clin Exp Pathol. (2015) 8:12276–83. 26722413 PMC4680358

[44] Sun C Zhang Z He P Zhou Y Xie X. Involvement of PI3K/Akt pathway in the inhibition of hepatocarcinoma cell invasion and metastasis induced by SASH1 through downregulating Shh-Gli1 signaling. Int J Biochem Cell Biol. (2017) 89:95–100. 10.1016/j.biocel.2017.06.006 28600143

[45] Zong W Yu C Wang P Dong L. Overexpression of SASH1 Inhibits TGF-beta1-Induced EMT in Gastric Cancer Cells. Oncol Res. (2016) 24:17–23. 10.3727/096504016X14570992647203 27178818 PMC7838734

