# Supplementary material for: Silencing SCAMP1-TV2 Inhibited the Malignant Biological Behaviors of Breast Cancer Cells by Interaction With PUM2 to Facilitate INSM1 mRNA Degradation
Source: Front Oncol. 2020 May 27;10:613. doi: 10.3389/fonc.2020.00613 (PMC7326047; doi:10.3389/fonc.2020.00613)
Supplement: Supplementary file 1 [file Data_Sheet_1.docx]

| Primer or Probe | Gene | Sequence (5'->3') or Assay ID |
| --- | --- | --- |
| Primer | SCAMP1-TV2 | F: CTTGTGGATGTGAGTGAC |
|  |  | R: GAGACCAGTAAGGGATGA |
|  | GAPDH | F:GGACCTGACCTGCCGTCTAG |
|  |  | R:TAGCCCAGGATGCCCTTGAG |
|  | PUM2 | F:CAACAGCAGCCAAGCACTAA |
|  |  | R:CCACTTCCAAAGCCAAGAGA |
|  | INSM1 | F:CAGTGTGCGGAGAGTCGTT |
|  |  | R:ACCTGTCTGTTTTCGGATGG |
|  | SASH1 | F:TGGAGGATTTGACTTGACGA |
|  |  | R:GAGATGCGAGAGGCAGTAGG |

Supplementary Table1 Primers used for detecting expression.

Supplementary Table2 Sequences of shRNA.

|  | Target sequence (5'->3') |
| --- | --- |
| SCAMP1-TV2 | GGATGTGAGTGACAGTTCA； |
| PUM2 | GAGGACTAATGCACTAACAGT |
| INSM1 | CACAAGTACTTCGAACGCAGC |
| SASH1 | GGAAAGAAAGAACCCTCTTCA |

Supplementary Table3 Primers used for ChIP experiments.

| Gene | Binding site or Control | Sequence (5'->3') |
| --- | --- | --- |
| SASH1 | PCR1 | F: GATGCGGGTATGTGTTGTCAC |
|  |  | R: GGTGCTAGTTTCCCCATCCA |
|  | PCR2 | F: TGGGCATTCAGGAGCCAAAA |
|  |  | R: CACAAGTAGTCCGTGTCGCT |
