# Supplementary material for: Silencing SCAMP1-TV2 Inhibited the Malignant Biological Behaviors of Breast Cancer Cells by Interaction With PUM2 to Facilitate INSM1 mRNA Degradation
Source: Front Oncol. 2020 May 27;10:613. doi: 10.3389/fonc.2020.00613 (PMC7326047; doi:10.3389/fonc.2020.00613)
Supplement: Supplementary file 2 [file Data_Sheet_2.docx]

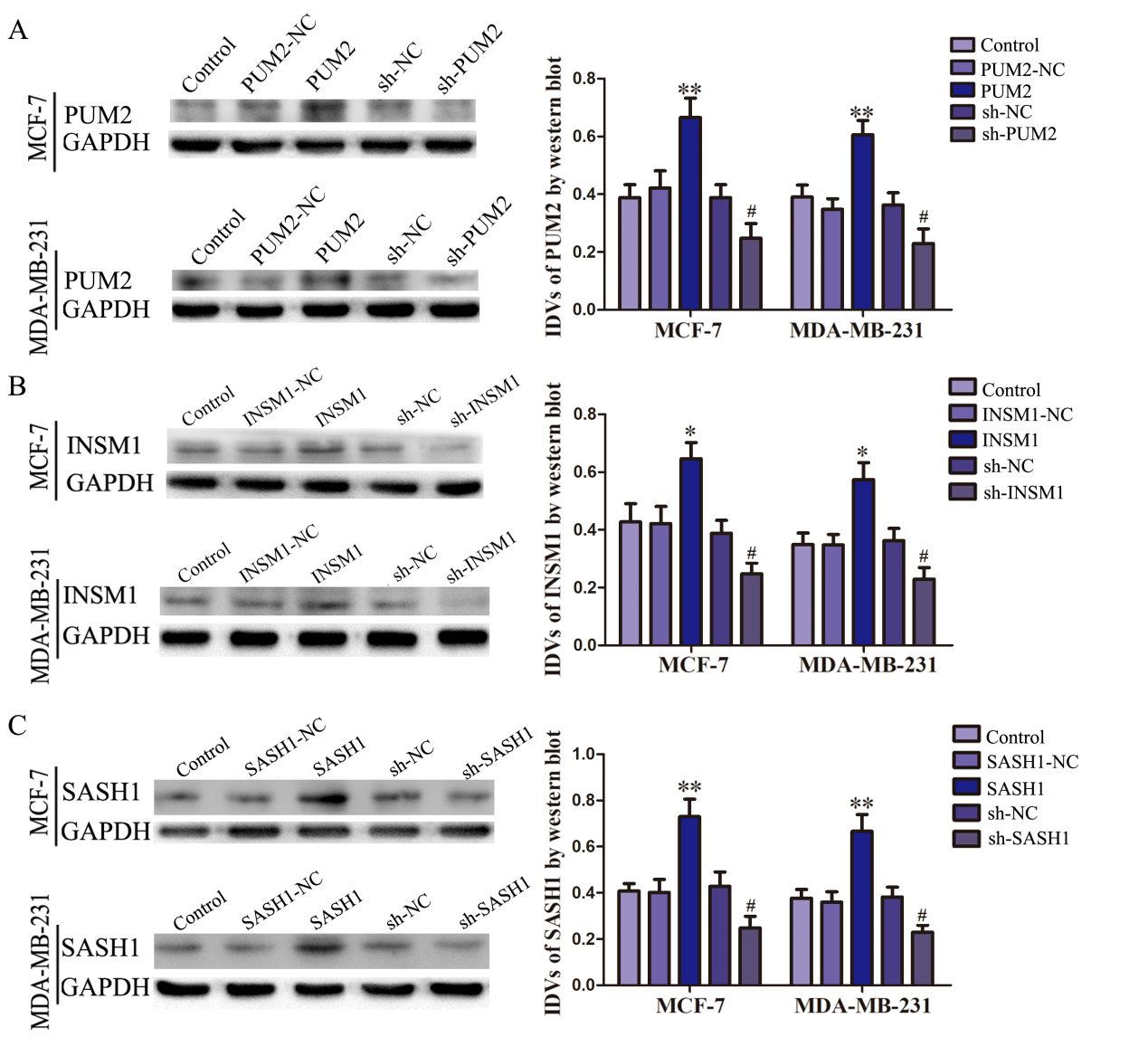


**Supplement Fig. 1 The expression efficiency of PUM2, INSM1 and SASH1 in MCF-7 and MDA-MB-231.**

(A) Expression of PUM2 after PUM2 over-expression and knockdown in MCF-7and MDA-MB-231 breast cancer cells. Data are presented as the mean ± SD (n=3, each group), ***P*<0.01 versus PUM2-NC group, ^#^*P*<0.05 versus sh-NC group. (B) Expression of INSM1 after INSM1 over-expression and knockdown in MCF-7and MDA-MB-231 breast cancer cells. Data are presented as the mean ± SD (n=3, each group), **P*<0.05 versus INSM1-NC group, ^#^*P*<0.05 versus sh-NC group. (C) Expression of SASH1 after SASH1 over-expression and knockdown in MCF-7and MDA-MB-231 breast cancer cells. Data are presented as the mean ± SD (n=3, each group), ***P*<0.01 versus SASH1-NC group, ^#^*P*<0.05 versus sh-NC group.


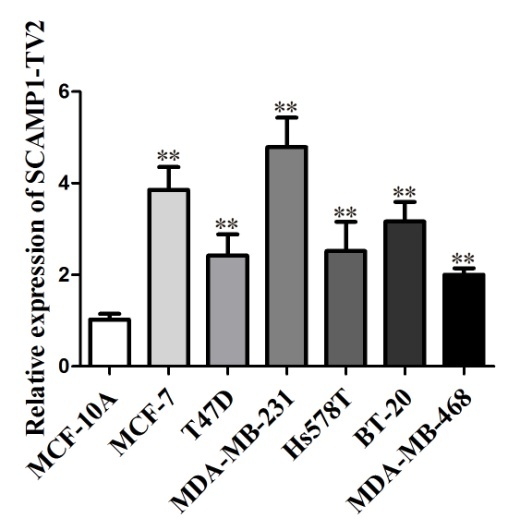


**Supplement Fig. 2 The expression of SCAMP1-TV2 in breast cancer cell lines.**

Expression levels of SCAMP1-TV2 in MCF7, T47D, MDA-MB-231, Hs578T, BT-20 and MDA-MB-468 cell lines. Data are presented as the mean ± SD (n=3, each group). ***P*<0.01 versus MCF-10A group.


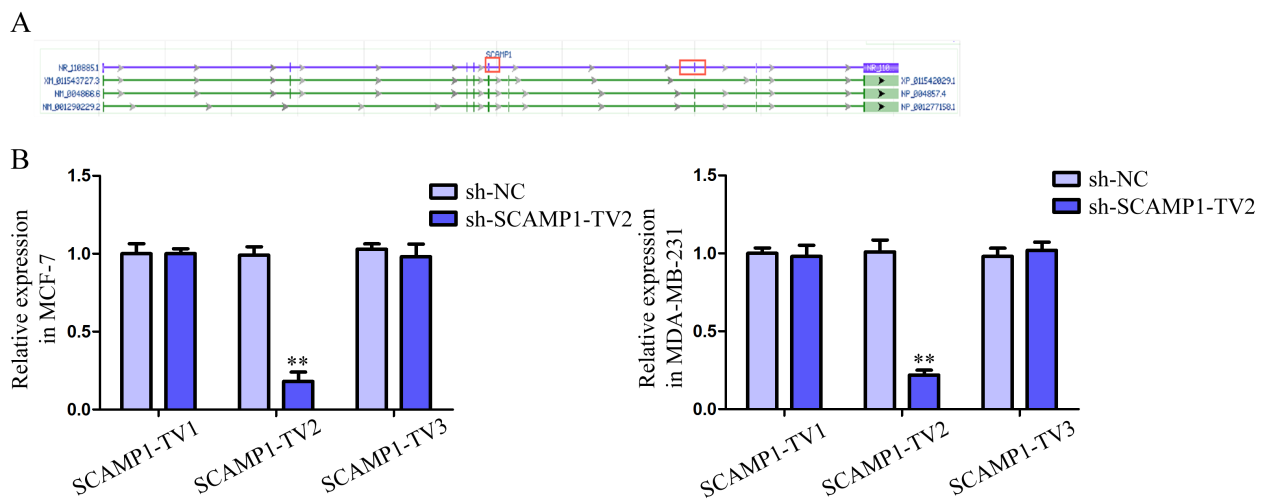


**Supplement Fig. 3 The expression of different transcripts of SCAMP gene after SCAMP-TV2 knockdown in MCF-7 and MDA-MB-231 cells.**

1. The picture of different transcripts of SCAMP gene, the red square box indicated the difference between the SCAMP-TV2 and other transcripts. (B) Expression of different transcripts of SCAMP gene after SCAMP-TV2 knockdown in MCF-7 and MDA-MB-231 cells. Data are presented as the mean ± SD (n=5, each group), ***P*<0.01 versus sh-NC group.


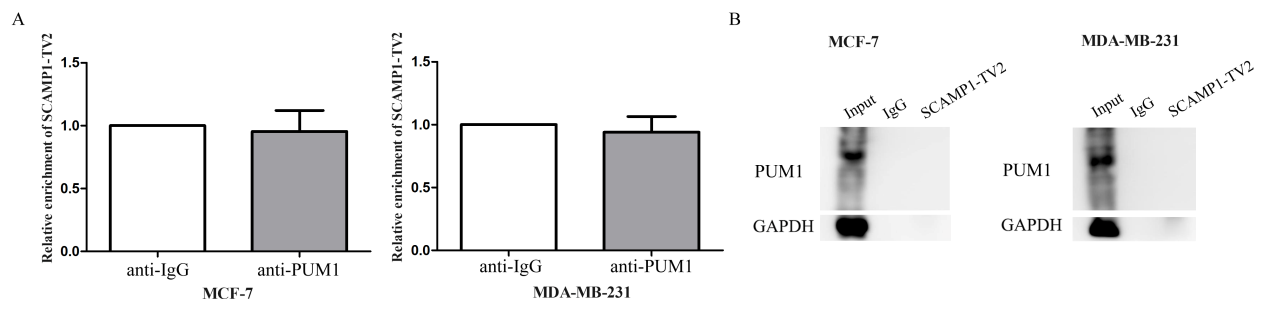


**Supplement Fig. 4 There was no binding site between SCAMP1-TV2 and PUM1.**

(A) Cellular lysates from MCF-7 and MDA-MB-231 cells were used for RNA immunoprecipitation with antibody against PUM1, SCAMP1-TV2 expression levels were detected using qRT-PCR. Data were presented as mean ± SD (n=3, each group). (B) Detection of PUM1 using western blot analysis in the sample pulled down by biotinylated SCAMP1-TV2 probe from MCF-7 and MDA-MB-231 cells.


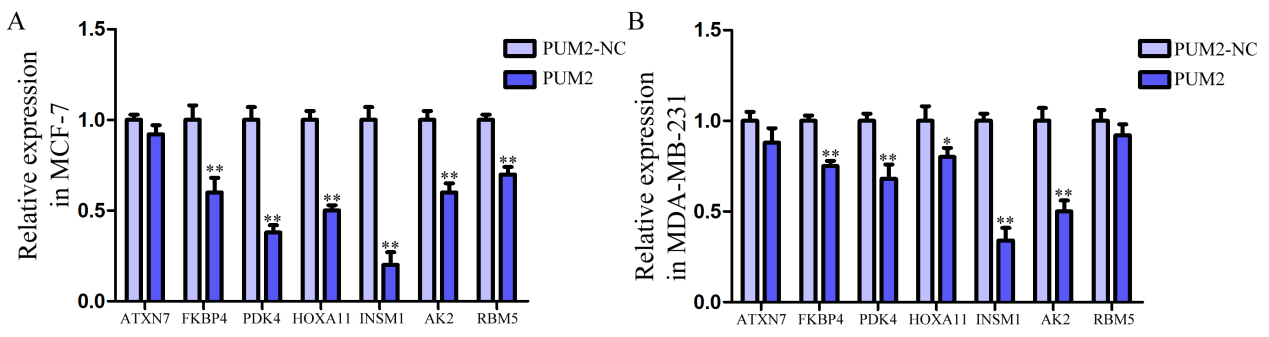


**Supplement Fig. 5 The expression of several mRNA after PUM2 over-expression in MCF-7 and MDA-MB-231 cells.**

Using the bioinformatics software, there are potential binding sites of PUM2 in 3’-UTR of several mRNA (ATXN7, FKBP4, PDK4, HOXA11, INSM1, AK2, RBM5). Expression of these mRNA after PUM2 overexpression in MCF-7 (A) and MDA-MB-231 cells (B). Data are presented as the mean ± SD (n=5, each group), **P*<0.05 and ***P*<0.01 versus PUM2-NC group.


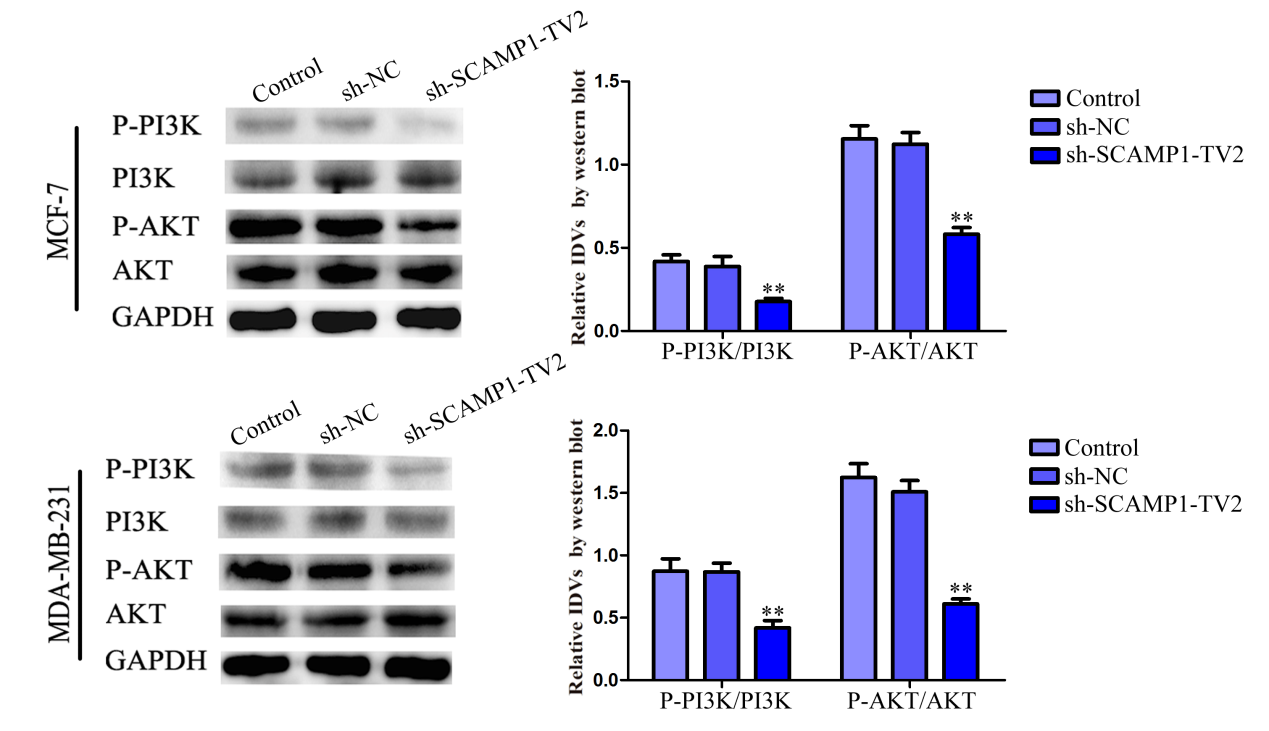


**Supplement Fig. 6 SCAMP1-TV2 knockdown on the activity of PI3K/AKT pathway in MCF-7 and MDA-MB-231 cells.**

Effects of SCAMP1-TV2 knockdown on the activity of PI3K/AKT pathway. The IDVs of PI3K, P-PI3K, AKT, P-AKT are shown using GAPDH as an endogenous control. Data are presented as the mean ± SD (n=3, each group), ***P*<0.01 versus sh-NC group.
